# Wound Healing and Antioxidant Properties of 80% Methanol Leaf Extract of *Verbascum sinaiticum* (Scrophulariaceae): An Ethiopian Medicinal Plant

**DOI:** 10.1155/2022/9836773

**Published:** 2022-09-08

**Authors:** Kalkidan Lulseged, Muluemebet Zinabu Akele, Alfoalem Araba Abiye, Besufekad Abebe, Solomon Assefa Huluka

**Affiliations:** ^1^Department of Pharmacology and Clinical Pharmacy, School of Pharmacy, College of Health Sciences, Addis Ababa University, Addis Ababa, Ethiopia; ^2^Faculty of Medical Sciences, University of Groningen, Groningen, Netherlands; ^3^Department of Pharmaceutical Chemistry and Pharmacognosy, School of Pharmacy, College of Health Sciences, Addis Ababa University, Addis Ababa, Ethiopia

## Abstract

Wounds account for severe morbidity, socioeconomic distress, and mortality around the globe. For several years, various herbal products are used to expedite and augment the innate wound healing process. In Ethiopian folkloric medicine, *Verbascum sinaiticum L*. (*V*. *sinaiticum*) is commonly applied as a wound-healing agent. The present study investigated the potential wound healing and antioxidant properties of hydroalcoholic leaf extract of *V. sinaiticum*. The 80% methanol extract, formulated as 5% (w/w) and 10% (w/w) ointments, was evaluated in excision and incision wound models using nitrofurazone and simple ointment as positive and negative controls, respectively. Parameters such as wound contraction, period of epithelialization, and tensile strength were determined. Moreover, its *in vitro* antioxidant property was evaluated using a DPPH assay. In the excision model, both doses (5% and 10% w/w) of the extract showed a significant (*p* < 0.001) wound healing efficacy compared to the negative control as evidenced by enhanced wound contraction rate and shorter epithelialization time records. In the incision model, the lower dose (5% w/w) ointment formulation of the extract exhibited the maximum increment in tensile strength (85.6%) that was significant (*p* < 0.001) compared to negative and untreated controls. Animals treated with 5% w/w ointment, furthermore, showed a significantly (*p* < 0.05) higher percentage of tensile strength than nitrofurazone treated ones. Moreover, the hydroalcoholic extract of the plant showed a noticeable free radical scavenging property. The result of the present study upholds the folkloric use of *V. sinaiticum* in the treatment of wounds.

## 1. Introduction

A wound is a disruption of the normal function and architecture of the skin that results in the interruption of epidermal layer integrity at the injury site [1]. A wound can be caused by insults to the tissue via physical, chemical, microbiological, or immunologic stimuli [2]. Mammals have *in-situ* capabilities of healing wounds through tissue regeneration and repair processes that occur after the onset of the lesion. This essential physiological response is executed by the collaboration of many cells and their products [3].

Wounds can be broadly classified as acute or chronic depending on physiology or the time it takes to heal [4, 5]. Chronic, nonhealing wounds and their treatment result in a considerable morbidity and socioeconomic loss, mainly in low- and middle-income countries (LMICs) [5]. Annually, close to 14 million people, where the majority of them are from LMICs, are estimated to suffer from this unavoidable event of life [6]. In common diagnosis, wound is still a challenge with its early and late complications [7]. Attributed to its economic and patient care impacts, wound has received a great deal of attention [8].

Antibiotics including topical preparations are available for treating wound [9]. Besides being expensive, these agents possess problems such as drug resistance and hypersensitivity, which can delay the wound healing process [10, 11]. Drugs used for wound treatment are partially effective and constitute less than 3% of the total drugs in clinical practice [12]. High costs and difficulties of performing clinical trials, however, have hampered the growth of new treatment options for wound management [10]. This, hence, urges the discovery of newer, affordable, safe, and effective wound healing agents to combat this problem.

Biologically active compounds from plants have played a vital role in discovering various modern medicines to treat disease conditions plaguing mankind [13]. Several medicinal plants [14–19] were scientifically proved to alleviate wounds. Over the last decade, different Ethiopian medicinal plants [12, 20–22] were also investigated for their wound healing efficacy in animal models and found to be active.

*Verbascum sinaiticum L*. (*V. sinaiticum*; Scrophulariaceae), colloquially known as “qetetina” (Amharic), is a biennial plant growing 60–150 cm in height. Various parts of this plant have folkloric uses in curing wounds (leaves), abdominal dropsy, anthrax, diarrhea, and fungal infections [23]. Moreover, this plant is endowed with pharmacologically proven hepatoprotective and antitrypanosomal properties [24, 25]. Wound healing is the main indication of the plant in Ethiopian folkloric medicine, albeit not proven pharmacologically. This study, thus, aimed at evaluating the *in vivo* wound healing and *in vitro* antioxidant properties of the 80% methanol extract of *V. sinaiticum* leaves.

## 2. Materials and Methods

### 2.1. Collection and Authentication of Plant Material

Fresh leaves of *V. sinaiticum* were collected from its natural habitat around Mersa district, Amhara Region, 490 km north of Addis Ababa, Ethiopia, in January 2020. The collected plant material was authenticated by a taxonomist (Mr. Melaku Wondafrash) at the National Herbarium, College of Natural and Computational Sciences, Addis Ababa University, where a voucher specimen (Y001) was deposited for future reference.

### 2.2. Extraction Procedure

The collected plant material was washed, air-dried under shade, and pulverized into a moderately coarse powder. Dried leaf powder (500 g) of *V*. *sinaiticum* was extracted by maceration with 80% methanol (1 : 10 ratio) for 72 h and the residue was remacerated twice. The filtrates of all portions were pooled into one vessel and concentrated using a rotary evaporator (BUCHI Rotavapor, Switzerland) at 40°C. The concentrated extract was frozen and dried in a lyophilizer at −50°C under reduced pressure. Finally, 76 g of dried 80% methanol extract (15.2% percentage yield) was obtained and transferred into a well-labeled vial to be stored in a refrigerator until further use.

### 2.3. Qualitative Phytochemical Analysis

The 80% methanol extract of *V. sinaiticum* was screened for the presence of active phytochemical constituents such as alkaloids, flavonoids, saponins, phenolic compounds, tannins, terpenoids, and anthraquinones using standard procedures [26, 27].

#### 2.3.1. Determination of Total Phenolic Content (TPC)

The total phenolic content of the extract was determined using the Folin–Ciocalteu's method [28]. The experiment was conducted in triplicate. The absorbance of the solution was measured at 760 nm using a UV spectrophotometer (Jenway Model 6500, England).

The total phenolic content was determined using a standard curve of gallic acid (*y* = 0.0073*x*−0.0951; *R*^2^ = 0.924).

#### 2.3.2. Determination of Total Flavonoid Content (TFC)

The total flavonoid content of the extract was measured using an AlCl_3_ complex-forming assay. Quercetin, a standard, was assayed using its different diluted concentrations (1, 0.50, 0.25, 0.125, and 0.065 mg/mL) via a method described in Kamtekar et al. [29]. The absorbance of the solution was recorded at 510 nm by a UV spectrophotometer (Jenway Model 6500, England). The same steps were followed for the extract (1 mg/ mL) and blank solution.

The total flavonoid content was determined using a standard curve of quercetin (*y* = 0.1329*x* + 0.0155; *R*^2^ = 0.924).

### 2.4. Experimental Animals

Animals used in this study were obtained from the animal house of the School of Pharmacy, Addis Ababa University. Healthy adult Wistar rats (*Rattus norvegicus*) of either sex (150–200 g and aged 3-4 months) were used to conduct dermal toxicity and wound healing activity studies. Female Swiss albino mice (25–30 g and 8–10 weeks of age), on the other hand, were used to conduct the oral acute toxicity study. Animals were housed in cages under standard conditions (22 ± 3°C, and 12 h light and dark cycles) and had free access to standard pellet diet and water. Animals were acclimatized to the laboratory conditions for a week before the experiment and they were handled according to the International Guidelines for the Use and Care of Laboratory Animals [30].

### 2.5. Ointment Formulation BP

The base ointment BP (British Pharmacopoeia) of the extract was prepared using wool fat, hard paraffin, cetylstearyl alcohol, and white soft paraffin with a formula described in the Pharmacopeia (Table 1) [31].

For the preparation of 80% methanol extract ointment, the levigation method was employed to make the ointment of uniform consistency, and smooth. Accordingly, plant extract ointment BP was prepared in 5% (w/w) and 10% (w/w) concentrations.

### 2.6. Toxicity Studies

#### 2.6.1. Acute Oral Toxicity Test

An acute oral toxicity study was done following the method described previously [32]. Accordingly, five female mice received a single dose (2000 mg/kg) of 80% methanol extract, orally. All mice were fasted for 4 h before and 2 h after the experiment and allowed water *ad libitum*. There were two phases of the study. In the first phase, a female mouse was weighed and dosed as explained above. The mouse was then observed for 24 h. Since no death was observed, the same dose was administered to the rest of the four female mice on the next day, for the phase two study. Finally, animals were observed for gross behavioral changes such as loss of appetite, hair erection, lacrimation, tremors, convulsions, and mortality for 14 days.

#### 2.6.2. Acute Dermal Toxicity Test

An acute dermal toxicity test was carried out using six female Wistar rats according to OECD guidelines [33]. Following a one-week acclimatization period, on both sides of each rat, a 500 mm^2^ area of fur was shaved from the dorsal area of the trunk before the experiment, one for the test substance and the other side for control.

In the respective shaved areas, 10% (w/w) of the extract and base ointment (control) was applied to fully cover shaved areas and both sides were covered by porous gauze and wrapped with a nonirritating bandage. Animals were then returned to their cages and provided with food and water. After 24 hours, the patches were removed, and the test substance and simple ointment were cleansed by distilled water. Approximately, at one hour, 24 h, 48 h, and 72 h, following the removal of the patches, the sites were examined for skin irritation reactions. The reactions, defined as erythema and edema, were evaluated according to the Draize dermal irritation scoring system (Table 2) for skin reactions ranging from 0 (No) to 4 (Severe) [34]. Moreover, the animals were observed for the development of any adverse skin reactions daily for 14 days.

To determine the potential skin irritation effect of the test substance, the primary irritation index (PII) was calculated. The test material was classified as per the scheme devised by Draize [35]. Accordingly, the substance was categorized as negligibly irritant (PII of <0.5), mildly irritant (PII of 0.5–1.9), moderately irritant (PII of 2–5), and severely irritant (PII of >5).

PII was calculated as follows:

(1) $$\begin{array}{c} \text{PII}=\frac{\sum \text{Erythema and Edema grade at }1,24,48,\text{ and }72\text{ hours}}{\text{No}.\text{ of test sites}\times 4\text{ scoring intervals}}. \end{array}$$

### 2.7. *In Vivo* Wound Healing Models

#### 2.7.1. Animals Grouping

Following the preparation of the wound site, animals were randomly assigned into four (excision model) and five (incision model) groups consisting of six rats each. In both models, group I rats were treated with a nonmedicated (base) ointment and served as a negative control. The second group (group II) rats received 0.2% (w/w) ointment of the standard drug, nitrofurazone, and served as a positive control. Rats in groups III and IV were treated with 5% and 10% w/w ointment formulation of *V. sinaiticum* extract, respectively. For the incision model, the fifth group was left untreated without applying any agents and served as an untreated control.

#### 2.7.2. Excision Wound Model

Before wound site preparation, each rat was anesthetized with an intraperitoneal (i.p.) injection of ketamine (50 mg/kg) and diazepam (1 mg/kg). The wound area was rubbed with 70% alcohol before depilating the outer skin fur from the dorsal thoracic area with a shaver. Approximately, 314 mm^2^ area was then marked with a thin permanent marker, 1 cm away from the vertebral column, and excised carefully using a sterilized surgical blade. Following their recovery (from anesthesia), animals were returned to their cages, and the day was considered day zero. After 24 h of wound creation (day one), treatment was commenced. Once daily, freshly prepared ointments were applied gently, according to their respective groups, to cover the entirety of the wounded area until complete healing of the wound was achieved [36].

*(1) Measurement of Wound Contraction*. The wound closure rate was assessed by measuring the contraction of the wound on every alternate postwounding day using a transparent paper and a permanent marker that was subsequently transferred into a graph paper. This measured surface area was used to calculate the percentage of wound contraction, taking the initial size of the wound as 100% as shown below [20]:

(2) $$\begin{array}{c} \text{\% Wound Contraction}=\frac{\text{Wound area on day 0}-\text{Wound area on day }n}{\text{Wound area on day 0}}\times 100\text{,} \end{array}$$

where *n* = the days where the measurement was taken (on days 2, 4, 6, 8, 10, 12, 14, 16).

*(2) Epithelialization Period Measurement*. Falling of dead tissue remnants without any residual raw wound was taken as an endpoint of complete epithelialization and days required for this process were taken as an epithelialization period (EP) [37]. Percent decrease in EP was calculated as follows:

(3) $$\begin{array}{c} \text{\% decrease in epithelialization periods}\left(\text{EP}\right)=\frac{\text{EP base}–\text{EP test}}{\text{EP base}}\times 100\text{.} \end{array}$$

#### 2.7.3. Incision Wound Model

In this model, animals were treated as described in the animal grouping section. On a wounding day, rats were anesthetized in the same way as the excision wound model. After wound area preparation, a 3 cm longitudinal incision was made on the paravertebral area of the skin and subcutaneous tissue at a distance of about 1.5 cm from the vertebral column with a sterile sharp surgical blade. Following complete hemostasis, the incised skin was then sutured 1 cm apart using a catgut suture with a curved needle (no. 11). The wound was left undressed and cleaned with a cotton swab as described by Perez et al. [38]. After 24 h of wound creation (on day 1), the respective topical formulation of the extract, standard, and base ointment was applied for nine consecutive days. Sutures were removed on day 8 postincision and the tensile strength of the healed wound was measured on the 10^th^ postwounding day using the continuous water flow technique [21].

*(1) Measurement of Tensile Strength*. Rats were anesthetized with ketamine (50 mg/kg, i.p.) and placed on the table using clippers. The two forceps were firmly applied 1 cm away from the healed tissue on the incised part of the skin onto the line facing each other. One is fixed and the other was connected to a freely suspended 1000 ml Volume IV bag through a string run over to the pulley. Water was allowed to slowly and continuously flow into a bag from tap water through the IV-line string until the wound just opened up and noted as an indirect measure of the tensile strength of the wound for an individual animal [39]. The percent strength was also calculated using the formulas shown below [20]:

(4) $$\begin{array}{c} \text{Tensile strength}\left(\text{TS}\right)\text{of extract}\left(\text{\%}\right)=\frac{\text{TSextract}-\text{TSbase}}{\text{TSbase}}\times 100\text{,}\\ \text{Tensile strength}\left(\text{TS}\right)\text{of standard}\left(\text{\%}\right)=\frac{\text{TSextract}-\text{TSstandard}}{\text{TSbase}}\times 100\text{,}\\ \text{Tensile strength}\left(\text{TS}\right)\text{of vehicle}\left(\text{\%}\right)=\frac{\text{TSbase}-\text{TSl.u.}}{\text{TSbase}}\times 100\text{,} \end{array}$$

l.u. = left untreated.

### 2.8. Antioxidant Activity

#### 2.8.1. DPPH Free Radical Scavenging Test

The DPPH (2,2-diphenylpicrylhydrazyl) free radical scavenging test is a quick, accurate, and inexpensive method of determining the free antioxidant capacity of different compounds [40]. In this assay, 50 *μ*L of various concentrations (10 g/mL, 5 g/mL, 2.5 g/mL, 0.625 g/ml, and 0.3125 g/mL) of the test samples was mixed with 5 mL of 0.004% methanol solution of DPPH. The mixture was incubated for 30 min at 37°C. After incubation, the absorbance of the mixture was read at 517 nm using UV spectrophotometer-UV-7804C.

Tests were carried out in triplicate. Percent inhibition was calculated as follows:

(5) $$\begin{array}{c} I\left(\%\right)=100\times \frac{\left(A^{0}-A^{s}\right)}{A^{0}}, \end{array}$$

where *A*^0^ is the absorbance of the control (containing all reagents except the test compound) and *A*^*s*^ is the absorbance of the tested sample. The IC_50_ value was calculated for all test samples using linear regression plots of concentration versus % of DPPH scavenged.

### 2.9. Statistical Analysis

Statistical analysis of the results was done by using SPSS software version 22.0. The experimental results obtained from both incision and excision wound models were expressed as mean ± SEM. Results were compared with the corresponding control groups using one-way ANOVA followed by the post-hoc Tukey test. The data were deemed statistically significant at *p* value < 0.05.

### 2.10. Ethical Considerations

Before experimenting with the animal models, the study protocol was ethically approved by the Ethical Review Committee of the School of Pharmacy, College of Health Sciences, Addis Ababa University. All the experimental procedures were conducted abiding by the International Guidelines for the Use and Care of Laboratory Animals [27].

## 3. Results

### 3.1. Phytochemical Screening

Preliminary phytochemical screening results revealed a strong and moderate presence of saponins and tannins, respectively, in the hydroalcoholic extract of *V. sinaiticum* leaves. Moreover, alkaloids, flavonoids, phenolic compounds, and terpenes were detected in the experimental plant.

#### 3.1.1. Total Flavonoid and Phenolic Content

**Figure 1:**
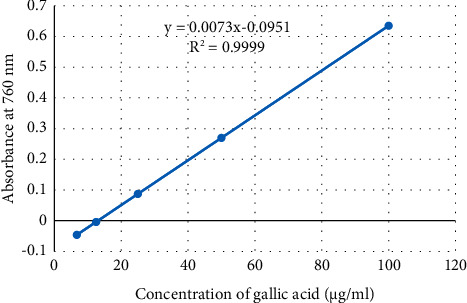
Calibration curve of gallic acid in the determination of total phenolic content of *Verbascum sinaiticum*.

**Figure 2:**
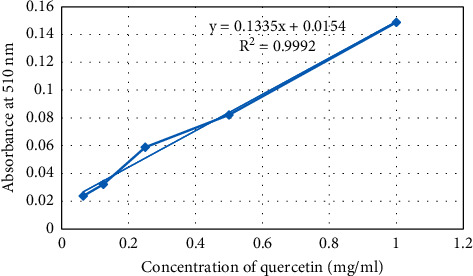
Calibration curve of quercetin in the determination of total flavonoid content of *Verbascum sinaiticum*.

The phenolic content of the extract is expressed as gallic acid equivalent (mgGAE)/g dry sample obtained from the calibration curve of gallic acid (Figure 1). The results in phenolic content determination assay showed that the extract of *V. sinaiticum* has a significantly high (167.95 mg of GAE/g dry sample) content of polyphenols per gram of dry extract.  Figure 2 shows the calibration curve of quercetin and the flavonoid content was found to be 0.168 mg of QE/g extract.

### 3.2. Toxicity Tests

#### 3.2.1. Acute Oral Toxicity Test

At a limit dose of 2000 mg/kg, administered orally, the 80% methanol extract did not result in mortality within the first 24 h and throughout the 14-day observation period. Morphological characteristics stated in the standard (fur, skin, eyes, and nose) appeared normal during the 14-day follow-up.

#### 3.2.2. Acute Dermal Toxicity Test

As per the Draize scheme, the application of the maximum ointment dose of the extract (10% (w/w) did not cause any sign of inflammation and edema. The PII value of the hydroalcoholic extract (≈0.009) was found to be close to base ointment (0.008), which explains the nonirritant (negligible irritancy) nature of the test sample.

### 3.3. Wound Healing Property

#### 3.3.1. Excision Model

**Figure 3:**
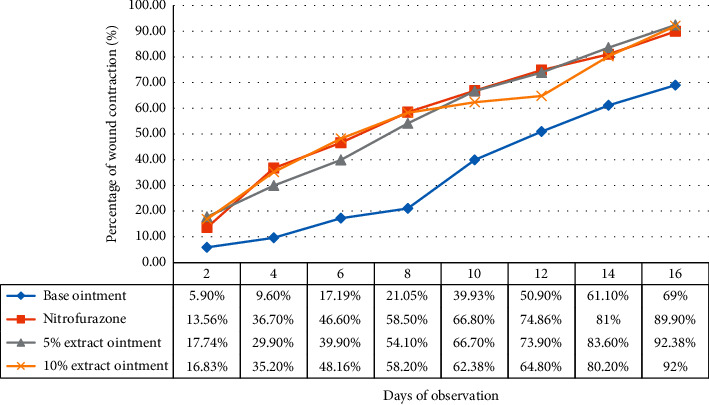
Percentage wound contraction of rats treated with hydroalcoholic extract of *Verbascum sinaiticum* in excision model. Values are expressed as mean ± SEM; *n* = 6.

*(1) Wound Contraction*. The daily progress of wound contraction produced by topical application of 5% and 10% w/w ointment of *V. sinaiticum* extract is summarized in Table 3. The extract and standard drug exhibited a similar onset of action in reducing wound size that was significant (*p* < 0.001) compared to the vehicle-treated group. Starting from the second day of treatment (day 2) to the end (day 16), both doses of the extract were able to significantly (*p* < 0.001) augment the wound contraction process of the excised wound. Despite the absence of a significant difference in wound healing activity between the 5% and 10% ointment doses of the extract, the higher rate of wound closure (92.38%) was observed with lower dose ointment (Figure 3). On day 16, the measured area of the wound was 94.3 ± 3.52, 33.5 ± 22.8, 23.9 ± 13.7, 24.9 ± 10.6 for simple ointment, nitrofurazone, 5%, and 10% ointment extracts treated groups, respectively.

Even though it failed to reach statistical significance, both doses of the extract exhibited a slightly superior wound healing effect than nitrofurazone. Percent wound closure of nitrofurazone, 5% extract, and 10% extract ointment showed a significant increment across time compared to the negative control. The relative order for the wound healing effect of different groups by percentage wound contraction was as follows: 5% extract >10% extract > nitrofurazone > base ointment.

*(2) Epithelialization Period*. The EP of all formulations tested in excision wounds is indicated in Table 4. The time required to completely heal the wound was significantly (*p* < 0.01) short in animals treated with extract ointments (5% and 10%) and nitrofurazone as compared to the ointment base treated group. There was no statistically apparent difference in the EP between the extract and the standard as well as between the two doses of the extract. However, 5% extract ointment showed the highest (14.7%) percentage reduction. On average, the period of epithelialization was 18.3, 18.0, and 18.6 days for standard drug, 5% (w/w) extract ointment, and 10% (w/w) extract ointment, respectively.

#### 3.3.2. Incision Model

In this model, a breaking strength (g) of 787.5 ± 51.53, 850.0 ± 106.06, and 900.0 ± 40.80 was recorded for the standard drug, 10% (w/w), and 5% (w/w), respectively, which was statistically significant (*p* < 0.001) compared to rats treated with the base ointment (485.6 ± 0.42) as well as untreated group (397.5 ± 0.42) (Table 5). Animals treated with a lower strength (5% w/w) of the extract showed the maximum (85.6%) percentage of tensile strength that was significantly higher (*p* < 0.05) than the standard drug, nitrofurazone. However, a statistically significant difference in mean tensile strength was not detected between the two formulations of *V. sinaiticum* (5% and 10%). Compared to the untreated group, the group treated with simple ointment base BP tended to increase the mean tensile strength by 22.6%.

### 3.4. Antioxidant Activity

**Figure 4:**
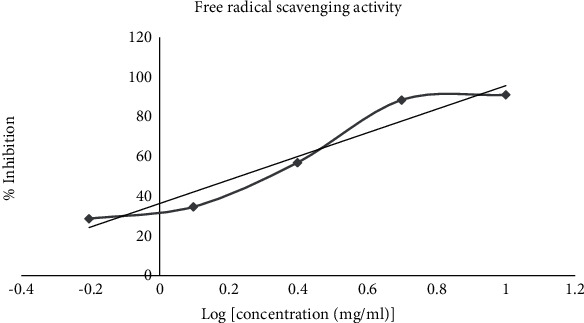
Percent inhibition of leaf extract of *Verbascum sinaiticum* as tested by the DPPH free radical scavenging test method.

The free radical scavenging property of the extract was determined in the DPPH assay and expressed using IC_50_ values. The observed change of color from pink to yellow in the test extract and standard solution confirmed that they have DPPH radical scavenging properties. The IC_50_ of the extract determined using linear regression was found to be 1.70 mg/ml (Figure 4).

## 4. Discussion

Despite the current advancement in medicine, wound management remains a clinical challenge. People used several plants and their derivatives as wound healing agents without any scientific shreds of evidence [18]. This study screened the Ethiopian plant, *V. sinaiticum*, which is used in traditional wound management. In pharmacological studies, *in vivo* screening continued to be indispensable for wound healing activity investigations mainly due to the intricacy of mammalian wound healing in damaged tissue [41]. Based on this notion, the present study was performed using excision and incision wound models in rats.

In the excision model, wound contraction enhances the closure of the defect by moving the edges of the wound towards the center. In this study, the progressive reduction of the wound area was monitored planimetrically. Accordingly, topical application of ointment formulations of *V. sinaiticum* showed a significant wound reduction compared to the vehicle-treated group. The pronounced reduction of wound size (≈ 92%) indicates the prohealing action of the plant [42]. Though further studies are required, stimulation of fibroblasts is plausible to be considered as the main mechanism of plant extract to facilitate wound healing. In wound sites, fibroblasts proliferate and enhance the production of collagen, the main constituent of the extracellular matrix [43].

The plant was tested for its effect on the epithelialization process, in which epithelial cells resurface the wound via the influence of growth factors [44]. The 5% w/w ointment preparation of the plant significantly reduced the epithelialization period. This comparatively short period of epithelialization could be attributed to the ability of phytoconstituents of the extract to enhance the synthesis of collagen and induce cellular proliferation [45]. Moreover, antioxidants that are present in herbal extracts are reported to promote epithelialization by controlling oxidative stress. Thus, the presence of secondary metabolites such as flavonoids that are endowed with a free radical scavenging activity can play a significant role in supporting the healing effect of the extract in excised wounds [46].

In DPPH antioxidant assay, the plant showed to have antioxidant activity with IC_50_ value of 1.70 mg/ml. The polyphenol content seems to be correlated with a dose-dependent scavenging potential of the plant. In effect, different phenolic compounds were isolated from the aerial parts of *Verbascum* species including quercetin, rutina harpagoside, protocatechuic acid, gentisic acid, *p*-coumaric acid, ferulic acid, salicylic acid, and rosmarinic acid [47].

In the incision wound model, both doses of the extract showed a significant increment of breaking strength compared to negative and untreated controls. This might in turn indicate the ability of the extract to enhance collagen maturation and partly improve the quality of the repaired tissue [48]. The lower strength (5% w/w) ointment of the extract exhibited a superior tensile strength percentage than 10% w/w strength, which is in agreement with the previous study [49]. A better wound healing effect of the lower dose observed in both models might be due to an increased concentration of certain irritant chemicals in 10% ointment that can cause recurrent inflammation in the wounded site.

Topical antimicrobial therapy is one of the most important interventions in wound care [50]. Accordingly, the enhanced wound healing activity of *V. sinaiticum* could also be attributed to its antimicrobial activity as it is previously reported by Yeabyo et al. [51] and proved efficacy against common wound pathogens. Possessing antibacterial activity could augment the wound healing effect of the plant.

Despite lack of pharmacological evidence, there are works of literature [52] reporting the traditional use of *V. sinaiticum* as an anti-inflammatory agent. Previous pieces of evidence showed that plants with anti-inflammatory properties can aid the wound healing process. Furthermore, plant-derived bioactive principles inhibiting inflammation can possess wound healing potential [53].

The observed wound healing potency of the plant could be due to the individual or synergistic functioning of phytoconstituents of *V. sinaiticum* to initiate innate mechanisms of wound healing activity. Previous studies revealed that saponins, flavonoids [54], and terpenoids [55] can accelerate numerous biological responses including antimicrobial, antioxidative, and anti-inflammatory activities. Hence, the synergistic effect of phytoconstituents in terms of these activities could significantly amplify the wound healing property of *V. sinaiticum*.

## 5. Conclusion

Findings of the current study showed that *V. sinaiticum* is endowed with a promising wound healing potency evidenced by its ability to improve wound contraction rate and increase wound breakage strength. Moreover, the safety profile of *V. sinaiticum* ointment formulation, augmented by its antioxidant property, enhances its wound healing efficacy. This study upholds the traditional use of the plant for the treatment of wound. Further studies on biofractionation are underway to isolate the active compounds. Considering further studies, this plant could serve as the potential source of effective lead compounds for wound healing.

## Figures and Tables

**Table 1:** Formula used for the preparation of base ointment.

Preparation of base (nonmedicated) ointment		
Ingredients	Master formula (in grams)	Reduced formula (in grams)
Wool fat	50	10
Hard paraffin	50	10
Cetylstearyl alcohol	50	10
White soft paraffin	850	170
Total	1000	200

**Table 2:** Classification system for skin reactions.

Erythema reaction	Score	Edema formation	Score
No erythema	0	No edema	0
Very slight erythema	1	Very slight edema	1
Well defined erythema	2	Well defined edema (edges of the area are well defined)	2
Moderate to severe erythema	3	Moderate edema (raising approximately 1 mm)	3
Severe erythema (beet redness) to scar formation	4	Severe edema (raising more than 1 mm and extended beyond the area of exposure)	4
The total possible score for primary irritation	8		

**Table 3:** Changes in excised wound size after topical application of the 5% and 10% extract ointment of *Verbascum sinaiticum* on rats.

Wound area (mm^2^) postwounding days								
Groups	2	4	6	8	10	12	14	16
BO	295.0 ± 2.54	283.8 ± 2.81	260.0 ± 2.3	247.9 ± 29.8	188.6 ± 2.00	153.9 ± 3.55	122.1 ± 2.49	94.3 ± 3.52
NFO (0.02%)	271.3 ± 12.8^a3^	198.5 ± 5.3^a2^	167.6 ± 11^a3^	130.1 ± 17.7^a3^	104.2 ± 16.5^a3^	78.9 ± 18.5^a3^	57.8 ± 22.39^a2^	33.5 ± 22.8^a3^
5% extract ointment	258.3 ± 3.5^a3^	220.0 ± 13.4^a3^	188.5 ± 13.3^a2^	144 ± 39.9^a3^	104.5 ± 11.05^a3^	81.89 ± 14.8^a3^	51.3 ± 18.25^a3^	23.9 ± 13.7^a3^
10% extract ointment	261.2 ± 6^a3^	203.2 ± 3.6^a3^	162.7 ± 5.57	131.2 ± 12.5^a3^	118.1 ± 3.3^a3^	99.2 ± 8.52^a3^	62.1 ± 14.06^a2^	24.9 ± 10.6^a3^

Values are expressed as mean ± S.E.M (*n* = 6); all superscripts indicate significance. a compared to control group; 2 = *p* < 0.01, 3 = *p* < 0.001; numbers from 2 to 16 indicate the day on which contraction rate measurement was taken; BO = base ointment; NFO = nitrofurazone ointment.

**Table 4:** Effect of topically administered ointment formulated from 80% methanol leaf extract of *Verbascum sinaiticum* on epithelialization period.

Groups	Epithelialization period (days)	% decrease in epithelialization period
BO	21.1 ± 0.08	
NFO (0.02%)	18.3 ± 1.30^a2^	13.04
5% extract ointment	18.6 ± 2.13^a2^	14.7
10% extract ointment	18.0 ± 1.57^a2^	11.8

Values are expressed as mean ± S.E.M (*n* = 6). All superscripts indicate significance. ^a^compared to control group; 2 *p* < 0.01; BO = base ointment; NFO = nitrofurazone ointment.

**Table 5:** Effect of topical application of 80% methanol leaf extract of *Verbascum sinaiticum* on breaking strength in an incision wound model.

Group	Breaking strength (g)	% Tensile strength
Left untreated	397.5 ± 0.42	—
BO	485.6 ± 0.42	22.16
NFO (0.02%)	787.5 ± 51.53^a3,b*3*^	76.1
5% extract ointment	900.0 ± 40.8^a3b*3*c1^	85.6
10% extract ointment	850.0 ± 106.06^a3b*3*^	75.2

Values are expressed as mean ± S.E.M (*n* = 6). All superscripts indicate significance. ^a^compared to control group, ^b^compared to untreated group, ^c^compared to the standard drug; 1 *p* < 0.05, *3* = *p* < 0.001; BO = base ointment; NFO = nitrofurazone ointment.

## Data Availability

The original dataset supporting the finding of the present study can be obtained from the corresponding author upon reasonable request.
